# Global Health Needs Modernized Containment Strategies to Prepare for the Next Pandemic

**DOI:** 10.3389/fpubh.2022.834451

**Published:** 2022-06-13

**Authors:** Krish Seetah, Hannah Moots, David Pickel, Marit Van Cant, Alessandra Cianciosi, Erin Mordecai, Mark Cullen, Yvonne Maldonado

**Affiliations:** ^1^Department of Anthropology, Stanford, CA, United States; ^2^Center for Innovation in Global Health, Stanford University, Stanford, CA, United States; ^3^Center for Population Health Sciences, Stanford School of Medicine, Stanford University, Stanford, CA, United States; ^4^Woods Institute for the Environment, Stanford, CA, United States; ^5^Department of Human Genetics, Oriental Institute Museum, University of Chicago, Chicago, IL, United States; ^6^Department of Classics, Stanford University, Stanford, CA, United States; ^7^Belgian American Educational Foundation (B.A.E.F), New Haven, CT, United States; ^8^Amsterdam School of Historical Studies, University of Amsterdam, Amsterdam, Netherlands; ^9^Department of Biology, Stanford University, Stanford, CA, United States; ^10^Faculty Development and Diversity, Global Health and Infectious Diseases, Department of Pediatrics, Stanford University, Stanford, CA, United States

**Keywords:** quarantine, history of medicine, anthropology, prevention, SARS-CoV-2, COVID-19

## Abstract

COVID-19 continues to be a public health crisis, while severely impacting global financial markets causing significant economic and social hardship. As with any emerging disease, pharmaceutical interventions required time, emphasizing the initial and continuing need for non-pharmaceutical interventions. We highlight the role of anthropological and historical perspectives to inform approaches to non-pharmaceutical interventions for future preparedness. The National Academy of Medicine, a not-for-profit, non-governmental US-based medical watchdog organization, published a key document early in the COVID-19 pandemic which points to inadequate quarantine and containment infrastructure as a significant obstacle to an effective pandemic response. In considering how to implement effective quarantine policies and infrastructure, we argue that it is essential to take a longitudinal approach to assess interventions that have been effective in past pandemics while simultaneously addressing and eliminating the negative socio-historical legacies of ineffective quarantine practices. Our overview reinforces the need for social equity and compassion when implementing containment.

## Introduction

Non-pharmaceutical interventions (NPIs) are highly effective at reducing disease transmission. Estimates suggest that COVID-19 cases in China in early 2020 would have been 67-fold higher without NPIs (1). Containment, an NPI involving both quarantine and isolation, is a first line defense in suppressing the spread of infectious disease. Quarantine is effective when used to restrict the movement of people and goods potentially exposed to infectious pathogens; isolation is effective when used to separate sick from healthy populations (2). However, evidence is conflicting on the feasibility of implementing and enforcing isolation and quarantine. During the 2020 outbreak in the USA, unclear guidelines and mixed messages about quarantine resulted in massive second and third waves of coronavirus infections (3, 4). Here we provide a longitudinal perspective to help distinguish between effective and ineffective utilization of quarantine; outline best practices, and caution against pitfalls observed in the historical record (5).

Between 1970 and 2000 the US Center for Disease Control and Prevention (CDC) reduced the number of quarantine centers from 55 to just seven (6). A 2006 National Academy of Medicine (NAM) report outlined inadequacies in US quarantine preparedness. Gaps were identified as a direct consequence of increased air travel and transmission rates of infectious disease. At that time, for the 120 million annual human transits through the nation's 474 ports, 25 quarantine stations were planned to screen goods, passengers, and animals (7). These figures illustrate the impossible expectations placed on these stations and the CDC who oversee them. Despite quarantine playing a critical role in the control of communicable diseases such as SARS, Ebola, and now COVID-19, adequate resources have not been devoted to maintaining an effective quarantine infrastructure; for example, only 20 stations are currently active. The NAM report brought to light the infrastructural and legal barriers to improving quarantine efficacy. Underscoring the NAM's conclusions are deeper seated socially embedded barriers that demonstrably reduce the effectiveness of containment.

## Longitudinal Socio-Economic Determinants Governing Containment

Efforts to understand the determinants of disease illustrate that social dimensions affect public and individual health in nuanced yet critical ways (8). The social sciences and humanities have an important role in studies of contemporary infectious disease, providing chronological, and social contextualization to inform global health policies (9). Fields such as archaeology and history are essential to identify practices that have been proven effective over time in the implementation of quarantine, as well as how these practices can be undermined by bias and xenophobia. Anthropology casts light on the cultural dimensions of disease (10). By providing insight on the relationships between disease and society, these disciplines could support targeted utilization of funds leading to improvements in well-being; however, this set of allied approaches remain underutilized for public health.

Modern ideas of quarantine are intrinsically linked to globalization. The term “quarantine” itself comes from the Italian for 40 days—*quaranta giorni*—the duration imposed on ships carrying goods and their crews before they could dock in Venice in the late 1400's.

**Figure 1:**
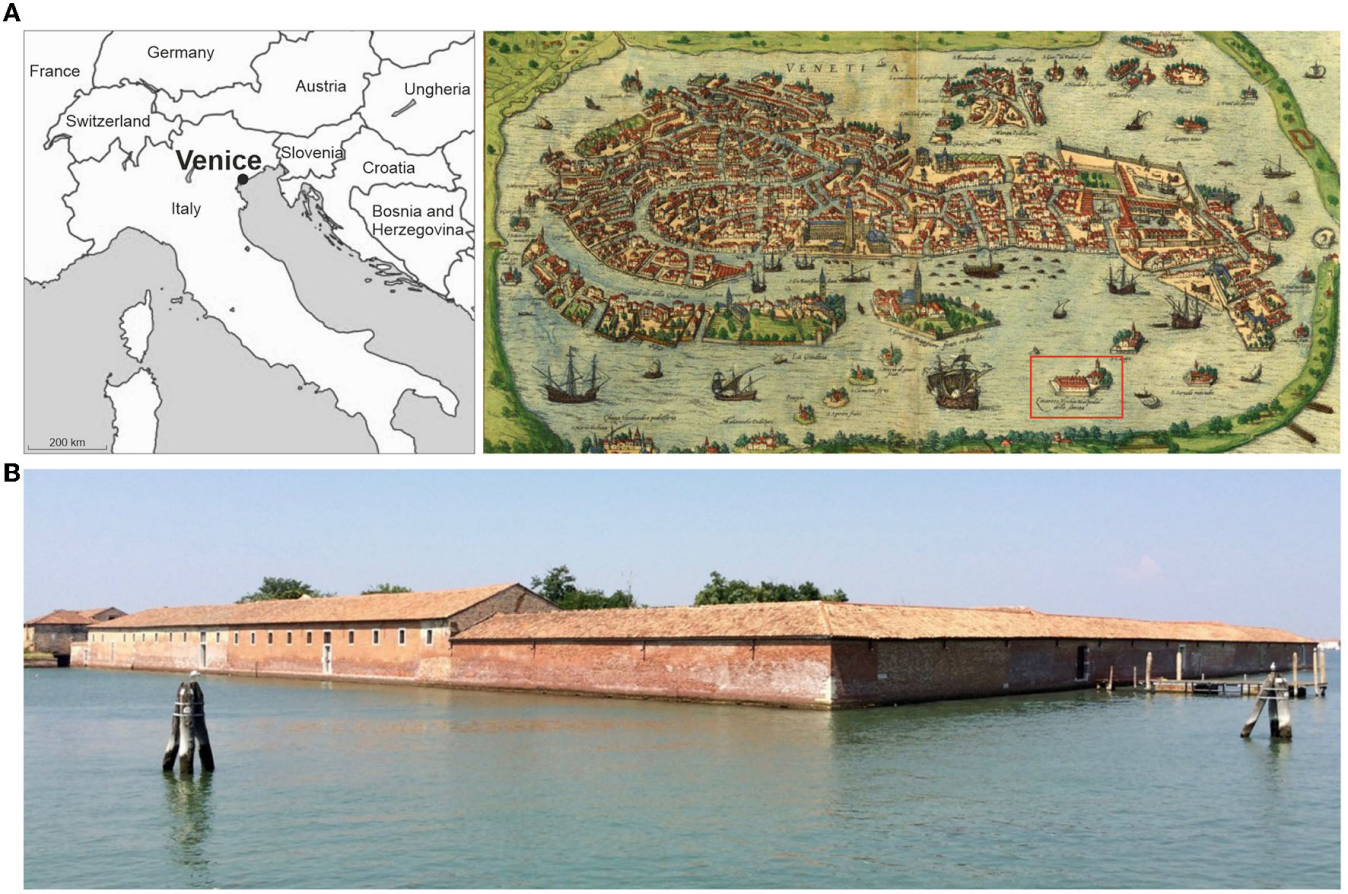
Venice, Italy: The Lazaretto Vecchio. **(A)** A sixteenth century map of Venice with the location of the Lazzaretto Vecchio (11) and **(B)** A recent image of the Lazzaretto Vecchio (photographed by H. Moots). The Lazzaretto Vecchio was established in 1423 about 2 km from Venice on a small island, close to modern Lido. It occupied a strategic location at the entrance to the Venetian lagoon from the Adriatic Sea. It was established some distance from the main inhabited islands, but still relatively closed to landing points so that goods could be easily transported, and passengers disembarked once quarantine was over.

The first maritime quarantine base was built on an island near Venice, Italy in 1423, known as Lazzaretto Vecchio (Figure 1), followed shortly after by Lazzaretto Nuovo. These earliest quarantine infrastructures and associated practices were developed from a growing understanding of infectious disease in the Middle Ages. During the Great Plague of 1347–1348, doctors recommended people vacate urban centers and ports for the countryside to avoid infection, believing *miasma* (“bad air”) caused disease. Although inaccurate, this theory did establish the connection between location and subsequent transmission of diseases (12).

Key infrastructural developments toward effective quarantine occurred during the sixteenth century in Italy where protocols were established to erect barriers in times of contagion. Ordinances published in 1576–1577 were a forerunner to social distancing, requesting that individuals stay at home and not go “wandering through the city and in other people's houses, mixing together with each other” (13). This multifaceted containment system, revolutionary for its time, was severely undermined through poor bureaucracy, corruption, and a lack of funds. The plague of 1575–1577 resulted in over 80,000 deaths in northern Italy (14).

Effective quarantining was implemented in England to prevent the spread of the bubonic plague from London to Sheffield in 1665. Local authorities imposed a 14-month quarantine on the village of Eyam, a stopping point between these two major cities, where a bale of flea-infested cloth had arrived from London. Imposing a quarantine, combined with regulations to confine cross-infection by performing religious services in open air and social distancing between families, ultimately proved effective. These measures contained the spread to other areas, but the death toll within the village could have been reduced by implementing “individual-level quarantine techniques” such as pest houses (15).

These tangible examples of quarantine are contrasted by intangible dimensions of quarantine, employed as a political tool to naturalize authority. British colonial policies for malaria mitigation in Nigeria in the 1940s, for example, were designed to reinforce pre-existing power dynamics. The implementation of “prophylaxis through segregation” was ultimately ineffective at preventing disease spread (16). Certain forms of quarantine, such as leper colonies, have also been linked to increased levels of stigmatization and lower quality of life for those quarantined and for proximal communities. Studies of leprosy in Greece, India, and Brazil have demonstrated how overt forms of quarantine increased social stigma toward the sick, encouraged inefficient use of public health resources, and resulted in lower levels of physical and socio-economic wellbeing for both infected and uninfected members of a community (17–19). When used for political control, quarantine is ineffective at thwarting disease spread and harmful to those targeted. Contemporary xenophobia around COVID-19 serve the same purpose as these past abuses, sowing division at a time that should compel cooperation.

## Gradients of Sickness and Risk

COVID-19 offers a stark example of the challenges faced by governments and health care professionals dealing with the way infections spread in the contemporary setting. Air travel has entirely transformed disease transmission. Quarantine has evolved from its utilization in the historic context, mainly to confine individuals or groups, to the extraordinary application to entire nations seen recently. Ethical considerations are particularly important with new forms of containment reliant on surveillance through mobile technology. The March 16th Imperial College report that galvanized governments in the UK and US into definitive action on COVID-19 situated the significance of a nuanced utilization of quarantine (2). Longitudinal and social lenses help leverage best-practice for improving future containment strategies, layered onto recommendations as proposed by the NAM, for example. A modernized conceptualization of containment that leverages evidence from the past should emphasize preparedness and be built on principles of ethical and compassionate utilization of quarantine. We propose three developments intrinsically dependent on engaging knowledge from the past.

The first task, tackling behavioral attitudes steeped in centuries of negative connotation, is arguably the most difficult. Pandemics come on the heels of and generate calamity that create social malaise, heighten a sense of urgency, and galvanize rapid action. These moments are fundamentally poorly timed for judicious decision-making. Thus, at the core of new quarantine measures is the need for foresight to anticipate pandemics and respond with compassion and humanity when they occur. History helps policy makers to recognize how public anxiety, stigmatization of at-risk groups, and politicized prejudice undermine efforts to suppress transmission of disease (20–22). Daily processes as part of quarantine need to be humane and responsive to maintaining psychological as well as physical health (23). One route to help tackle inherited bias is through considerate language. Terms such as “social distancing” and “shelter in place,” as forms of personal and local containment, show that modern implementations of quarantine as a concept need not be as draconian as practices in the past.

Second, policy must address the need for more effective surveillance of goods. Control of disease transmitted through cargo, not people, catalyzed the inception of quarantine as we know it today. Cargo remains a significant route through which diseases are transmitted across international borders (24). Measures are necessary that can respond in times when disruption occurs and be implemented within existing supply chains to identify and suppress transmission.

Finally, logistical measures can also benefit from adopting longitudinal and social perspectives to assist with future proofing. The current spread of new COVID-19 variants reveals the crucial role of international standards in travel strategies, combining a reinforced surveillance with contact tracing and testing optimization (25). Quarantine need not be viewed as an either/or option, but rather gradients of sickness and risk, adopting a nuanced response in specific ways tailored to individual diseases and the context in which the disease is to be treated (26). What are the parameters for transmission as understood in real-time or from similar diseases, and how can we operationalize quarantine interventions to respond to the specific epidemiology involved? An “agile and compassionate quarantine” should liaise closely with transport agencies, facilitating rapid response mechanisms that support reporting of disease directly to quarantine units. Underpinned by the need to be humane and aligning with principles of One Health, a modernized quarantine has to consider implementation in low-income settings to promote *global* well-being. Improved quarantine measures have been proposed after other recent pandemics (27). However, containment is rarely considered within the context of preparedness but rather as a last resort; a notion rooted in history. Effective preparedness is unlikely to be achieved without a thorough assessment of both past and present experiences. The COVID pandemic, characterized by new variants (28, 29) and the effects of uneven distribution of vaccines or drugs to counteract them (30), has showcased the need to reconsider and better align the use of quarantine, combined with systematic utilization of new tools, to enhance containment strategies. Modeling studies demonstrate the value of integrating quarantine and testing to reduce transmission of secondary infections (31–33). Distinguishing between the initial and subsequent utilization of quarantine has also proven important. Long periods of containment restricting travel were appropriate early in the pandemic when case numbers were asymmetrical between nations. However, later in the pandemic, research suggests that travel quarantine durations could be radically reduced, and in some cases, a short quarantine with testing may be as effective as an outright travel ban, thanks to the effectiveness of contact tracing and testing strategies (33).

## Conclusion

An action plan will require clinical, environmental, engineering, humanities, and social science expertise in dialogue with policy institutions and bodies such as the CDC and WHO. Subsequently, a rationalized universal overview of quarantine practice, drawing evidence from the large number of natural experiments taking place now and over the course of history, can be aligned to identify and share best practice that is applicable at a local, regional, or national level. These proposals are ambitious. However, they are necessary, and appropriately timed to update a powerful tool proven to be effective in combating the spread of infection. We have reached a critical juncture. Zoonotic diseases such as Ebola and coronavirus have exposed nations to lethal outbreaks of infectious disease. Unfortunately, these episodes have also laid bare the extent to which local, national, and international communities were unprepared for a pandemic of this scale in the modern day. The trauma COVID-19 has inflicted on the world will be relegated to memory. Nevertheless, copious research, alongside current experiences, signal the economic and social hardship from future pandemics. We cannot miss the opportunity to learn from the largest quarantine exercise in human history, nor to contextualize the present moment through longitudinal evidence. Quarantine became a social necessity in the past due to comparable factors that impact the modern world. This point should galvanize the political will and financial investment needed to modernize quarantine through research and policy initiatives. However, quarantine will be most effective once: (1) health disparities among high-risk populations are reduced, helping to create healthy communities; (2) the trust of all communities is engendered to promote participation in mitigation strategies, and by (3) strengthening public health infrastructure to support better quarantine. We inhabit a globe where disease in one part of the world can now appear almost simultaneous in another location; this can only be mitigated if containment is universal and sustainable. Re-casting quarantine as primarily concerned with promoting and maintaining healthy communities, rather than segregating those who present with disease, depolarizes the utility of quarantine, and invigorates an incredibly powerful tool in our arsenal against infectious disease.

## Data Availability Statement

The original contributions presented in the study are included in the article/supplementary material, further inquiries can be directed to the corresponding author/s.

## Author Contributions

All authors listed have made a substantial, direct, and intellectual contribution to the work and approved it for publication.

## Funding

Funding was provided by the Stanford Interdisciplinary Graduate Fellowship (HMM), Andrew W. Mellon Foundation Dissertation Fellowship (DP), the European Union's Horizon 2020 research and innovation programme under the Marie Skodowska-Curie grant agreement No 897004 (AC), and the Belgian American Educational Foundation (MV). EM was funded by the National Science Foundation (DEB-2011147 with the Fogarty International Center), the National Institutes of Health (R35GM133439), the Stanford Woods Institute for the Environment, the Stanford Center for Innovation in Global Health, and the Stanford King Center for Global Development. KS received funding from the Institute for Research in the Social Sciences, the Lang Fund for Environmental Anthropology, and from the Center for Innovation in Global Health Seed Grants.

## Author Disclaimer

Views are those of the authors and are not necessarily those of their institutions or funders.

## Conflict of Interest

The authors declare that the research was conducted in the absence of any commercial or financial relationships that could be construed as a potential conflict of interest.

## Publisher's Note

All claims expressed in this article are solely those of the authors and do not necessarily represent those of their affiliated organizations, or those of the publisher, the editors and the reviewers. Any product that may be evaluated in this article, or claim that may be made by its manufacturer, is not guaranteed or endorsed by the publisher.
